# Weighing the Risks: The Impact of Body Mass Index on Outcomes After Frozen Elephant Trunk Aortic Arch Repair

**DOI:** 10.3390/medicina62050973

**Published:** 2026-05-16

**Authors:** Tim Walter, Joseph Kletzer, Tim Berger, Salome Chikvatia, Magdalena Bork, Sophie Kunzmann, Mario Lescan, Stoyan Kondov, Aleksandar Dimov, Martin Czerny, Maximilian Kreibich, Dalibor Bockelmann

**Affiliations:** 1 Department of Cardiovascular Surgery, Heart Centre Freiburg University, University Medical Centre Freiburg, 79106 Freiburg, Germany; 2 Faculty of Medicine, University of Freiburg, 79106 Freiburg, Germany; 3 Staff Unit for Medical Strategy and Cooperation, University Clinic Freiburg, 79106 Freiburg, Germany

**Keywords:** frozen elephant trunk (FET), BMI, obesity, weight, arch surgery

## Abstract

*Background and Objectives*: This study aimed to evaluate the impact of body mass index (BMI) on post- operative outcomes in patients undergoing aortic arch repair with the frozen elephant trunk technique (FET). *Materials and Methods*: A total of 387 patients who underwent an FET procedure between 04/2014 and 11/2024 were retrospectively analyzed. Patients were divided into four groups according to BMI: underweight (BMI < 18.5, *n* = 12) normal weight (BMI: 18.5 to <25, *n* = 150), overweight (BMI: 25 to <30, *n* = 154), and obese (BMI: ≥30, *n* = 71). Patient characteristics and clinical outcomes were compared across groups. Multivariable Cox regression, interaction analysis, and restricted cubic spline modelling were performed using R (Version 4.4.3). *Results*: Interaction analysis revealed BMI-dependent effect modification for several predictors. Insulin-dependent diabetes mellitus was associated with increased mortality only in patients with BMI < 25 kg/m^2^ (interaction *p* = 0.003). Transfusion of packed red blood cells (PRBCs) also showed a significant interaction with BMI (*p* = 0.016), with a stronger effect in patients with BMI < 25 kg/m^2^, although significant in both strata. Moreover, cross-clamp time demonstrated a BMI-dependent interaction (*p* = 0.047), with numerically higher mortality hazards in overweight patients (BMI > 25 kg/m^2^), but without statistically significant subgroup effects. Spline analysis indicated a non-linear, threshold-based association between overall mortality and BMI but does not reach statistical significance. Kaplan–Meier analysis showed no significant difference in 5-year survival among BMI categories. *Conclusions*: BMI should not be used as a primary risk stratification tool for survival after an FET procedure. Rather, attention should be paid to comorbid conditions and intraoperative factors that interact with BMI. For patients with lower BMI (<25 kg/m^2^), optimizing glycemic control and minimizing transfusion may improve outcomes. Data suggests that a reduction in cross-clamp time may be particularly beneficial in patients with higher BMI (>25 kg/m^2^). Future studies should aim to clarify the impact of BMI on outcomes after FET, particularly in the context of patient selection and perioperative optimization strategies.

## 1. Introduction

The frozen elephant trunk (FET) procedure has evolved as a common and effective treatment in patients with thoracic aortic pathologies involving the aortic arch, with favourable postoperative outcomes in patients with acute or chronic aortic dissections, aortic aneurysms, or penetrating aortic ulcers [1,2]. The role of body mass index (BMI) in influencing surgical outcomes after cardiac and aortic procedures has been a subject of investigation given the global rise in obesity. Recent studies point to mixed findings, with some reporting no adverse effect of BMI on early major adverse events or mid-term survival after major surgery; some others do report adverse effects. Studies in cardiac surgical populations confirm that increasing obesity is associated with longer ICU stays, increased ventilator time and higher rates of ICU readmission. Obesity also impairs postoperative mobilization due to reduced exercise tolerance, increased risk of venous thromboembolism, and higher rates of infection, all of which can further delay recovery and discharge [3,4,5,6,7]. However, obesity is a known risk factor for cardiovascular morbidity and may complicate perioperative management, necessitating focused research to clarify its impact on outcomes in patients undergoing total aortic arch replacement using the FET technique.

Our aim was to evaluate the impact of BMI on postoperative outcomes and overall mortality in patients undergoing aortic arch repair with the FET technique.

## 2. Materials and Methods

Patients and follow-up protocol: Between 04/2014 and 11/2024, 459 patients underwent FET total arch replacement in one aortic centre currently performing over 60 total aortic arch procedures per annum (as of 2024). Four BMI groups were defined as the following: underweight patients with a BMI < 18.5 kg/m^2^, normal-weight patients with a BMI between 18.5 kg/m^2^ and 25 kg/m^2^, overweight patients with a BMI ranging between 25 kg/m^2^ and 30 kg/m^2^, and obese patients with a BMI over 30 kg/m^2^. The primary outcome measure was all-cause mortality at 5 years. After excluding patients with missing endpoint data (patients with estimated but not clearly provided preoperative weight, *n* = 72), 387 patients were included in the final analysis. Patients were followed up with, with a median follow-up of 3.85 [first quartile: 0.79; third quartile: 5.45] years. All patients were routinely followed up with after six months, twelve months and yearly thereafter in our dedicated aortic clinic. Computed tomography angiography (CTA) scans were done preoperatively, before discharge, during every follow-up visit, and when clinically warranted.

Surgical approach and technique: Our institutional technique for the FET procedure involves either full or upper hemi-sternotomy with routine right axillary artery cannulation for arterial inflow. Concomitant procedures (valve, aortic root, or coronary surgery) are performed during systemic cooling to 25 °C [8]. Myocardial protection is achieved with cold-blood cardioplegia or a beating-heart strategy [9,10,11]. Bilateral cerebral perfusion is standard, with selective use of trilateral antegrade perfusion via left axillary cannulation. Therefore, preoperative imaging includes CTA of the supra-aortic vessels and Circle of Willis.

FET implantation is routinely performed in zone 2 using the short (100 mm) Thoraflex™ hybrid graft (Terumo Aortic, Inchinnan, UK). In aneurysmal disease, the stent graft is oversized by 10% at the distal landing zone; in aortic dissection, oversizing is avoided, and sizing follows institutional criteria. Cerebrospinal fluid drainage is not routinely employed.

Indication for additional cardiac procedures: Any concomitant or additional surgical interventions, when indicated, were performed in accordance with the recommendations outlined in the 2014 European Society of Cardiology (ESC) Guidelines on the diagnosis and treatment of aortic disease and the 2024 European Association for Cardio-Thoracic Surgery and the Society of Thoracic Surgeons (EACTS/STS) Guidelines for diagnosing and treating acute and chronic syndromes of the aortic organ [2,12]. These procedures included, but were not limited to, aortic root replacement, valve repair or replacement, and coronary artery bypass grafting, and were executed following the guideline-endorsed strategies for optimal operative timing, surgical technique, and perioperative management to ensure adherence to contemporary evidence-based standards of care.

Data collection and definition of parameters: Data were collected retrospectively, relying on our prospectively maintained aortic database. Acute aortic dissection was defined as symptom onset fewer than 14 days before hospital admission and was classified as chronic if symptoms had occurred >14 days beforehand. The TEM classification was used to categorize aortic dissections (type A, type B, type non-A non-B) [2]. Postoperative stroke was defined as an acute neurological deficit of vascular origin confirmed by clinical assessment and neuroimaging, occurring after surgery and persisting for >24 h. Initiation of renal replacement therapy (hemodialysis or continuous renal replacement therapy) for acute kidney injury following surgery, regardless of perioperative renal function, was defined as postoperative dialysis. Postoperative paraplegia was characterized as new onset of complete motor and sensory loss in the lower extremities confirmed by neurological examination, occurring after surgery. We describe postoperative tracheotomy as the surgical or percutaneous creation of a tracheal stoma for airway management performed after the index surgery. ICU stays (days) are defined by the number of calendar days from admission to the ICU postoperatively until discharge from the ICU. In parallel, hospital stay (days) was defined as the number of calendar days from hospital admission for surgery until discharge from the hospital. Death from any cause occurring during the index hospitalization for the FET surgery was summarized as in-hospital mortality. We characterized a new FET thrombus in the first follow-up CTA as radiologically confirmed thrombus formation within the FET prosthesis detected on the first scheduled postoperative CTA scan. Elapsed time, measured in years, from the date of index surgery to the date of the first follow-up CTA-scan was defined as time to first follow-up CTA (years).

Statistical Analysis: The Feldschuh–Enson formula was used to estimate patients’ blood volume. Based on the DuBois formula for body surface area and percentage deviation from ideal weight, it was calculated as 100× (actual weight-ideal weight)/ideal weight.

Univariable and multivariable time-to-event analyses were performed using Cox proportional hazards regression models. For univariable analyses, each potential predictor was individually evaluated for its association with the outcome of interest. Results were summarized as hazard ratios (HRs) with 95% confidence intervals and corresponding *p*-values. For multivariable model building, stepwise variable selection based on Akaike’s information criterion (AIC) was applied to identify a parsimonious set of predictors. From this final model, adjusted HRs and 95% CIs were reported. Proportional hazards assumptions were checked using Schoenfeld residuals and corresponding tests derived from a standard Cox model. The ratio of predictors to outcome events was approximately 1:10, and therefore, no additional penalization or shrinkage methods to reduce overfitting were applied.

In multivariable analyses, these selected variables were entered simultaneously to identify independent associations. The proportional hazards assumption was assessed where appropriate. To explore whether the effect of predictors varied by subgroups (e.g., by body mass index), interaction terms between candidate predictors and the subgroup variable were included in Cox regression models. Interaction *p*-values were reported, and interaction plots were generated when evidence for effect modification was suggested (interaction *p* < 0.1). Survival probabilities were estimated using Kaplan–Meier methods. Survival curves were compared across groups using the log-rank test as appropriate. Graphical representations included 95% confidence intervals, risk tables, and cumulative event plots. Follow-up time was displayed in years, and group-specific survival probabilities were visualized. To flexibly model the association between continuous predictors and the outcome, restricted cubic spline functions were incorporated into Cox regression models. This approach allowed for assessment of potential non-linear relationships between continuous exposures and hazard of the outcome, adjusting for a set of predefined covariates where necessary. Predicted log-hazard ratios and corresponding confidence intervals were plotted across the range of the continuous exposure, with a clinically relevant reference value indicated. Histograms of the exposure distribution were overlaid to aid interpretation.

## 3. Results

Patient characteristics: In our study, differences reached statistical significance for age distribution (*p* = 0.019), hyperlipidemia (*p* = 0.011), arterial hypertension (*p* = 0.037) and diabetes mellitus (*p* = 0.045). Also, the distribution of heritable thoracic aortic disease (HTAD) differed significantly between the four groups, with more patients having HTAD with a BMI < 25 kg/m^2^. All patient characteristics are summarized in Table 1.

Aortic details: Previous cardiac interventions were frequent among the patients, with a comparable distribution across the different weight groups. A significantly higher rate of previous hemi-arch replacement was observed in underweight patients (*p* = 0.049). In addition, a significant difference between the four groups was observed regarding the occurrence of type B aortic dissection, with normal-weight patients showing a higher rate. (*p* = 0.03). All other aortic details can be found in Table 2.

Surgical details: Table 3 summarizes the intraoperative characteristics. Overall, surgical parameters were comparable across the weight groups, except for a statistically significant difference in the number of implanted conduits, with higher rates in the underweight group. A further significant difference was noted specifically in the transfusion of packed red blood cells (PRBCs). In contrast, transfusion rates of other blood products (fresh frozen plasma, platelets) showed no statistically significant variation between the groups. Across the four BMI groups, leaner patients exhibited a significantly lower estimated total blood volume and a higher percentage of transfused blood volume relative to their total blood volume, particularly for PRBCs and fresh frozen plasma (FFP) (*p* < 0.001). 

**Figure 1:**
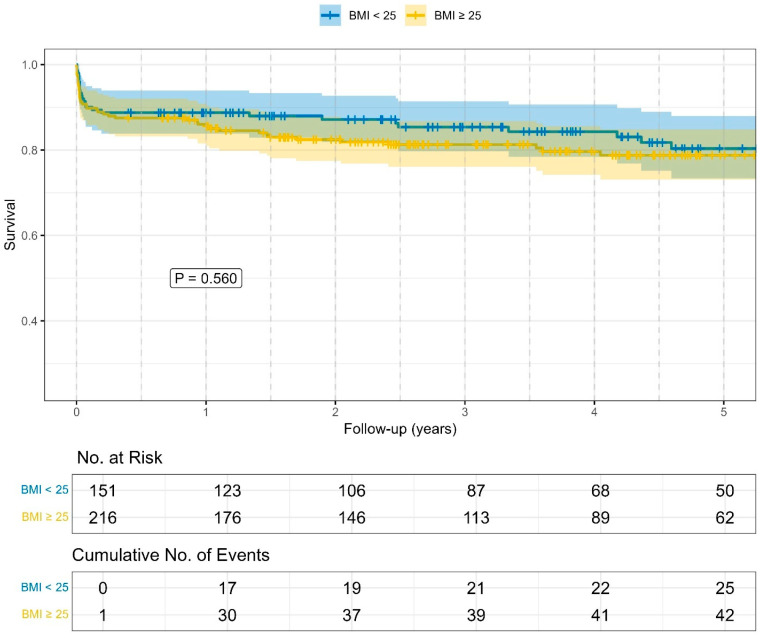
The Kaplan–Meier curve of the overall survival of the two BMI groups following frozen elephant trunk total arch replacement over 5 years. Log rank: *p* = 0.560. Time in years.

Outcome characteristics: The data are presented in Table 4. Significant differences between the four BMI groups were observed for hospital length of stay (*p* = 0.032) and the detection of new thrombus formation on the initial CTA scan (*p* = 0.003). In contrast, Kaplan–Meier analysis (Figure 1) revealed no significant difference in overall survival between the BMI groups (log rank: *p* = 0.560).

**Figure 2:**
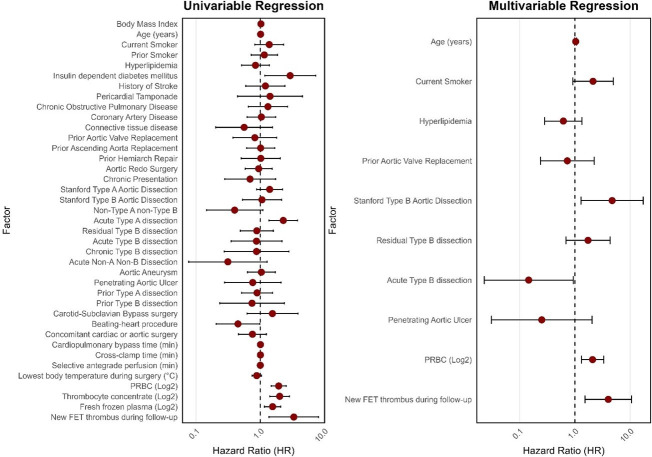
Univariable and multivariable analyses shown as forest plots. Factors are plotted across the hazard ratios; the CIs are marked by vertical lines.

Regression analysis: Type B dissection remained independently associated in the multivariable regression (HR: 4.722; CI 1.298–17.178). Also, new FET thrombus during follow-up was independently associated with increased hazard (HR: 4.022; CI 1.521–10.638). Finally, PRBC transfusion (log2-transformed; effect per doubling of the transfused volume) showed statistically significant hazard ratios. (HR: 2.089; CI 1.316–3.315). Models incorporating variables that were statistically significant in the univariable regression were explored but not retained in the final multivariable analysis according to the selection strategy outlined in Section 2. The full results of the regression analysis are shown as forest plots in Figure 2.

**Figure 3:**
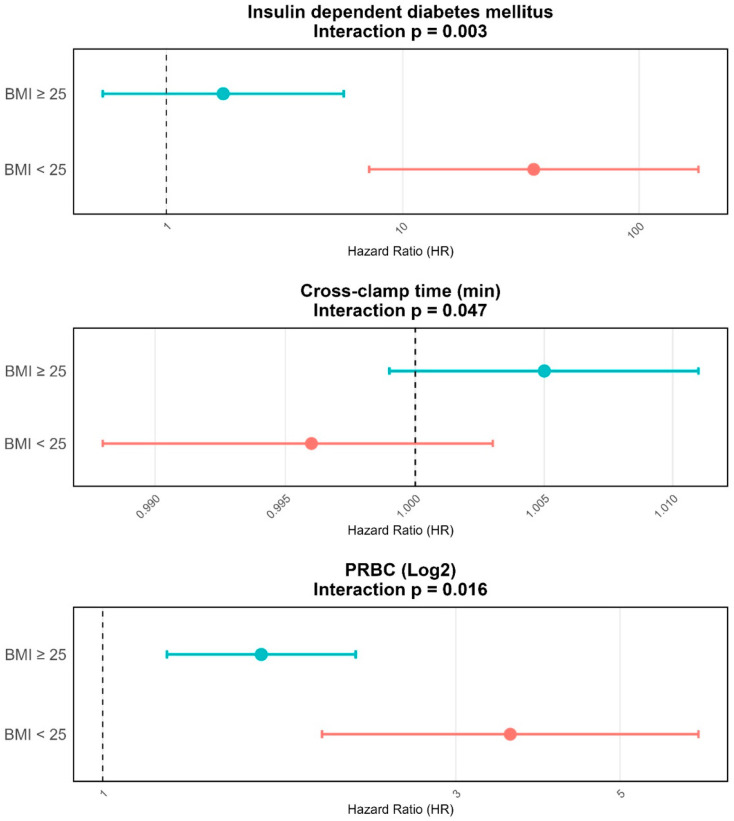
Interaction analysis for significant interaction *p*-values. When evidence for effect modification was suggested, an interaction was identified (interaction *p* < 0.100). Interaction analysis of the effect of different independent factors in different BMI subgroups on the hazard of death. Patients with BMI > 25 kg/m^2^ are represented with blue colour; the patients with BMI < 25 kg/m^2^ are coloured red. Horizontal lines represent the CI.

To explore whether the effect of predictors varied by subgroup we used an interaction analysis. Insulin-dependent diabetes mellitus showed a heterogeneous effect in patients based on their BMI. (*p* = 0.003). It reached statistical significance in the subgroup of BMI < 25 kg/m^2^, while the other subgroup (BMI > 25 kg/m^2^) did not. Further, the interaction term for transfusion of PRBC was statistically significant (*p* = 0.016). In stratified analyses according to BMI, both subgroups demonstrated a significant association with increased mortality risk. However, the magnitude of this association was greater among patients with a BMI < 25 kg/m^2^, who exhibited a more pronounced increase in the hazard of death compared with those with a BMI ≥ 25 kg/m^2^, while the effect remained statistically significant in both strata. For cross-clamp time, the interaction with BMI was statistically significant (*p* = 0.047), indicating that the association between ischemic duration and mortality differed across BMI subgroups. However, the subgroup-specific estimates did not reach statistical significance. Numerically higher mortality hazards with longer cross-clamp times were observed in overweight patients (BMI > 25 kg/m^2^; HR > 1), whereas the association appeared neutral in normal-weight patients (BMI ≤ 25 kg/m^2^; HR ≈ 1). The corresponding results are presented in Figure 3.

**Figure 4:**
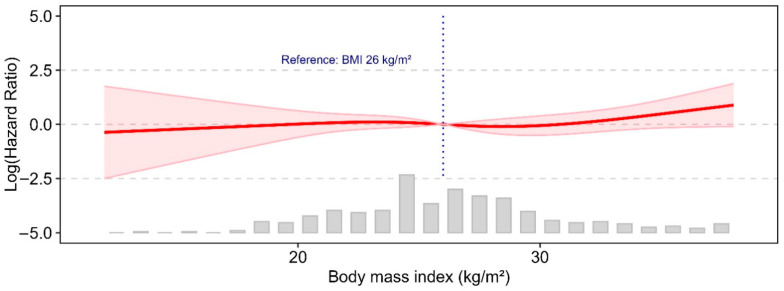
Restricted cubic spline analysis of the relationship between body mass index and overall mortality. Predicted log-hazard ratios and corresponding confidence intervals plotted across the range of the continuous exposure, with a clinically relevant reference value indicated. The threshold line is body mass index at 26 kg/m^2^.

Spline analysis of the relationship between body mass index and overall mortality after aortic arch repair reveals a non-linear, threshold-based association. There was a slight increase above the threshold of BMI 26, which does not meet the conventional threshold for statistical significance. The cubic spline regression can be seen in Figure 4.

## 4. Discussion

Our study’s most important findings can be summarized as follows: (I) BMI should not be used as a primary risk stratification tool for survival after total aortic arch repair following the FET procedure. Rather, attention should be paid to comorbid conditions and intraoperative factors that interact with BMI. (II) For patients with lower BMI (<25 kg/m^2^), optimizing glycemic control and minimizing transfusion may improve outcomes. (III) Additional research evidence is needed to better identify patients with higher mortality risk. The cardiovascular risk profile observed in our cohort shows strong parallels with findings from other studies on patients undergoing total aortic arch replacement. In line with the existing literature, our data confirm that a higher BMI is generally associated with a greater prevalence of traditional cardiovascular risk factors such as hypertension, dyslipidemia, and diabetes mellitus, all of which may complicate perioperative management and affect long-term outcomes [13,14]. Conversely, when focusing on patients with HTAD, our results are consistent with prior reports indicating that this population often exhibits BMI values below 25 kg/m^2^ [15]. This lower BMI distribution in HTAD patients is commonly attributed to the genetic and phenotypic characteristics of connective tissue disorders. Importantly, the relatively larger proportion of HTAD patients in our underweight subgroup may not only account for the numerically younger age observed in this group but also highlight the distinct pathophysiological background compared to non-HTAD patients [15,16]. It should also be noted that our cohort represents a distinct, high-risk population with prolonged circulatory arrest times and extensive aortic reconstruction, in whom BMI-associated effects may differ from conventional cardiac surgery. Taken together, these findings suggest that BMI cannot be interpreted solely as a marker of general cardiovascular risk; rather, it reflects the interplay between acquired risk factors in overweight and obese individuals and the genetic predispositions that characterize the HTAD population. The incidence of prior cardiac and aortic surgeries was high in this collective among all weight groups, with a tendency for more prior aortic surgeries in the lower-weight groups. This trend may be explained by the higher incidence of HTAD patients in this group as well. After all, our data is in line with prior reports that redo total arch repair using the frozen elephant trunk is a feasible and common surgical procedure [17,18,19,20]. The need for a secondary FET implantation is well reflected in the high number of patients with chronic aortic dissections here. In addition, our data is in line with the current EACTS aortic guidelines giving the FET implantation its place also in Type B and Non-A Non-B aortic dissection [2]. Because we did not see statistically significant differences among the weight groups, our decision-making for the FET implantation is most likely driven by radiographic and clinical (symptomatic) markers and not by BMI assessment.

In our cohort, the prevalence of prior cardiac and aortic surgery was notably high across all BMI categories, with a numerical trend toward more prior aortic interventions in lower-weight patients. This observation can likely be explained by the overrepresentation of individuals with HTAD in this group, as these patients typically require surgical intervention at a younger age and are prone to multiple reoperations over the course of their disease. The high rate of redo procedures and the frequent application of total arch replacement with the FET technique in our series reflect both the chronic disease burden in this patient population and the increasing establishment of FET as a standard and reproducible approach for complex aortic pathologies. Importantly, the substantial proportion of chronic dissections underscores the ongoing need for secondary FET implantation as part of staged or stepwise management strategies. These findings agree with current EACTS and STS guidelines, which highlight the role of the FET technique not only in acute type A but also in type B and non-A, non-B dissections, thereby reinforcing its versatility in clinical practice [2]. From a broader perspective, the lack of significant differences in FET utilization across BMI groups suggests that surgical decision-making in complex aortic disease is predominantly shaped by anatomical and radiological considerations, as well as by clinical symptomatology, rather than by patient body habitus. Collectively, our results add to the growing evidence base supporting the role of FET as a central tool in contemporary aortic surgery, bridging the treatment of diverse patient populations across the BMI spectrum.

Surgical characteristics reveal a considerable proportion of concomitant cardiac and aortic root procedures in this cohort. In line with our own previous findings and the current literature, we confirm that concomitant interventions, particularly aortic root replacement, can be performed safely in the context of FET implantation [8,21,22]. Notably, the implantation rate of valved conduits differed significantly across the four weight groups, with higher frequencies among both overweight and underweight patients. Several factors may explain this observation. In overweight patients, the increased incidence of valved conduit implantation could be related to a higher prevalence of acquired cardiovascular comorbidities, such as hypertension and aortic valve disease, which predispose to root pathology requiring replacement. Conversely, the higher rate in underweight patients is likely influenced by the greater proportion of individuals with HTAD in this group. Our data further highlight the relevant role of the beating-heart technique during FET implantation, the advantages of which have been confirmed both in our own experience and in reports from the Hannover group [9,10,23]. Interestingly, no differences in surgical times were observed across the BMI categories, indicating that patient body habitus alone does not appear to significantly impact surgical exposure or the technical aspects of operative access, particularly to the subclavian arteries. Notably, however, we found a statistically significant variation in the transfusion of PRBC between the BMI groups, with lower transfusion requirements in overweight and obese patients and a greater need in patients with lower BMI values. This observation is consistent with findings reported in other series, including cohorts of acute type A aortic dissection [13,24]. A plausible explanation may lie in the reduced physiological blood reserves of underweight patients, leaving them more vulnerable to relative blood loss and anemia during complex procedures. Conversely, patients with higher BMI often have larger circulating blood volumes, providing a greater buffer against intraoperative blood loss and thus reducing the relative need for transfusion. Our data regarding the estimated blood volume and the percentage of the estimated blood volume transfused was statistically significant for PRBC, platelets, fresh frozen plasma and the total transfused blood products among the four groups. It showed a clear inverse relationship between BMI and transfusion percentage: patients with BMI > 30 kg/m^2^ received a median of 6.93% (IQR 3.09–11.61%) of their estimated blood volume as PRBC, while those with BMI < 18.5–25 kg/m^2^ received much higher percentages (44.72% and 41.1% respectively), with a statistically significant difference. Leaner patients (BMI < 18.5–25 kg/m^2^) also received significantly higher percentages of FFP and the total amount of transfused blood products. This pattern indicates that even when using ideal body weight for blood volume estimation (as we assumed), underweight and normal-weight patients receive a substantially higher proportion of their estimated blood volume as transfusion compared to overweight and obese patients; these findings are consistent with large cohort studies showing that lower BMI is associated with increased transfusion rates and higher transfusion volumes relative to blood volume while higher BMI is protective against transfusion [24,25]. In transfusion practice these results highlight the importance of considering BMI and the method of blood volume estimation when interpreting transfusion requirements. Transfusion thresholds and strategies may need further individualization for patients at the extremes of BMI [24,26].

Our outcome parameters reflect the heterogeneous disease spectrum within the cohort. Importantly, no statistically significant differences were observed in major clinical outcomes, including in-hospital and long-term mortality. This result is likely attributable to the largely comparable intraoperative variables and the similar underlying disease characteristics across the groups. The frequency of tracheostomy was higher in overweight patients, although this difference did not reach statistical significance, most likely due to the limited sample size. By contrast, both in-hospital and ICU length of stay were longest in the underweight group, underscoring the limited physiological reserve and increased perioperative vulnerability of these patients. These findings are in line with the current literature [3,27,28,29].

New thrombus formation within the FET stent graft was detected in 7.4% of the overall cohort. Adding to this we made an interesting observation within the subgroup of underweight patients, where new thrombus formation occurred in 5 out of 12 patients (42%). Naturally, because of this small sample size these findings should be interpreted with caution and should be regarded as hypothesis-generating rather than conclusive.

While the precise risk factors for thrombus development remain to be fully elucidated, our findings may provide an initial indication that low BMI, potentially in conjunction with previously reported factors such as female gender, could be associated with an increased predisposing variable [30,31,32]. Crucially, thrombus formation emerged as a strong predictor of in-hospital mortality in our Cox regression analysis, further emphasizing its clinical relevance [33]. These results highlight a pressing need for future research focused on understanding the determinants of thrombus formation after FET implantation and on developing targeted strategies for risk mitigation in vulnerable subgroups, particularly underweight patients with bigger sample sizes.

Our data also suggest that BMI does not independently predict long-term mortality in this surgical population. However, interaction analyses reveal clinically relevant modifiers of risk within specific BMI strata. In patients with BMI < 25 kg/m^2^, insulin-dependent diabetes mellitus and PRBC transfusion (log2) are associated with an increased hazard ratio for overall mortality, indicating that metabolic comorbidity and perioperative transfusion requirements confer additional risk in leaner patients. The significant interaction term for cross-clamp time and BMI (*p* = 0.047) indicates that patient subgroups responded differently to prolonged ischemic duration. Although subgroup-specific effects were not statistically significant, the hazard for mortality was numerically higher in overweight patients (BMI > 25 kg/m^2^), suggesting a possible trend toward greater vulnerability with longer cross-clamp times. This exploratory pattern might reflect underlying differences in cardiac reserve, myocardial perfusion, or susceptibility to reperfusion injury. In contrast to the so-called ‘obesity paradox’ described in some cardiac surgery cohorts [34], our results point to context-specific risk modulation during aortic arch repair and warrant confirmation in larger, prospective studies. The spline analysis suggests a potential non-linear association between BMI and postoperative mortality after aortic arch repair using the FET procedure; however, these findings should be interpreted with caution, as the analysis was not statistically definitive. Rather than indicating a firm threshold effect, the results may point to a possible trend toward higher risk at the lower end of the BMI spectrum, particularly among underweight patients, who may be more vulnerable because of frailty, malnutrition, or relevant comorbidities. At higher BMI values, the association appears less pronounced, although a modest increase in risk may become more apparent at the extreme upper end of the distribution. Overall, these observations should be regarded as exploratory and hypothesis-generating rather than as evidence of a clinically established cut-off [34,35,36].

Given the potential for residual confounding related to unmeasured aspects of frailty, sarcopenia, and nutritional status, the observed associations should be interpreted with caution and confirmed in studies with more comprehensive covariate assessment.

The absence of direct evidence linking BMI to mortality in FET cohorts supports the need for further investigation. Nevertheless, the integration of BMI into preoperative risk assessment may be justified given its established prognostic value in other cardiac and vascular surgical populations. Future studies should aim to clarify the impact of BMI on outcomes after FET, particularly in the context of patient selection and perioperative optimization strategies.

### Limitations and Strengths

Our study is limited by its small sample size and retrospective nature. Figure 3’s first panel shows a very wide 95% confidence interval, reflecting a relatively small number of patients in this subgroup. Therefore, the precision of the estimate is limited, and the results should be interpreted with caution. In addition, the potential for residual confounding related to unmeasured factors such as frailty, sarcopenia, and nutritional status cannot be excluded and may partially explain the observed BMI-associated outcomes. However, this investigation contributes valuable knowledge on weight-specific differences in patients requiring FET total arch replacement.

## 5. Conclusions

In patients undergoing total aortic arch repair with the FET procedure, body mass index may be associated with postoperative outcomes in a non-linear manner, with a potential increase in risk at higher BMI values, particularly above approximately 26/kg m^2^. However, given the limitations of the present analysis, these findings should be interpreted as exploratory rather than definitive. BMI should therefore not be considered an isolated or linear determinant of postoperative risk stratification. Instead, overall risk assessment should place greater emphasis on established clinical risk factors, including renal dysfunction, severe atherosclerosis, and malperfusion syndromes, as well as on intraoperative variables such as cardiopulmonary bypass and cross-clamp times, which appear to be more strongly related to adverse outcomes after FET, including in redo procedures. In patients with lower BMI, attention to perioperative optimization, including glycemic control and judicious transfusion management, may be reasonable. Although further studies are required to confirm any causal relationship.

## Figures and Tables

**Table 1:** Patient characteristics.

Characteristic						
Number of Patients	Overall N = 387 ^1^	≥30 N = 71 ^1^	30 > x ≥ 25 N = 154 ^1^	25 > x ≥ 18.5 N = 150 ^1^	<18.5 N = 12 ^1^	*p*-Value ^2^
Age (Years)	66 (58, 74)	64 (58, 72)	66 (57, 74)	69 (59, 76)	60 (52, 66)	0.019
Current Smoker	91 (24%)	18 (26%)	35 (23%)	34 (23%)	4 (36%)	0.700
Prior Smoker	165 (43%)	33 (48%)	73 (48%)	54 (37%)	5 (45%)	0.200
Hyperlipidemia	139 (37%)	35 (51%)	59 (39%)	43 (29%)	2 (18%)	0.011
Hypertension	327 (86%)	63 (89%)	138 (90%)	118 (80%)	8 (73%)	0.037
Diabetes mellitus	12 (3.1%)	4 (5.8%)	6 (3.9%)	1 (0.7%)	1 (9.1%)	0.042
History of Stroke	44 (12%)	4 (5.8%)	21 (14%)	16 (11%)	3 (27%)	0.110
Pericardial Tamponade	12 (3.1%)	0 (0%)	8 (5.2%)	4 (2.7%)	0 (0%)	0.200
Renal Failure	42 (11%)	8 (11%)	15 (9.7%)	19 (13%)	0 (0%)	0.600
Chronic Obstructive Pulmonary Disease	37 (9.7%)	7 (9.9%)	9 (5.8%)	20 (14%)	1 (9.1%)	0.120
Coronary Artery Disease	115 (30%)	20 (28%)	53 (34%)	40 (27%)	2 (18%)	0.500
Bicuspid Aortic Valve	14 (3.7%)	1 (1.4%)	5 (3.3%)	8 (5.5%)	0 (0%)	0.500
Heritable Thoracic Aortic Disease	29 (7.6%)	2 (2.8%)	8 (5.2%)	16 (11%)	3 (27%)	0.011

^1^ Median (Q1, Q3); *n* (%); ^2^ Kruskal–Wallis rank sum test; Fisher’s exact test.

**Table 2:** Preoperative characteristics.

Characteristic						
Number of Patients	Overall N = 387 ^1^	≥30 N = 71 ^1^	30 > x ≥ 25 N = 154 ^1^	25 > x ≥ 18.5 N = 150 ^1^	<18.5 N = 12 ^1^	*p*-Value ^2^
Prior Coronary Artery Bypass Grafting	11 (2.9%)	1 (1.4%)	4 (2.6%)	5 (3.4%)	1 (9.1%)	0.500
Prior Aortic Valve Replacement	41 (11%)	7 (10%)	13 (8.6%)	20 (14%)	1 (9.1%)	0.500
Prior Mitral Valve Replacement	4 (1.1%)	0 (0%)	1 (0.7%)	3 (2.0%)	0 (0%)	0.500
Prior Ascending Aorta Replacement	110 (29%)	21 (30%)	44 (29%)	40 (27%)	5 (45%)	0.600
Prior Hemiarch Repair	48 (13%)	11 (16%)	20 (13%)	13 (8.8%)	4 (36%)	0.049
Type A Dissection	145 (37%)	28 (39%)	64 (42%)	49 (33%)	4 (33%)	0.400
Acute	68 (47%)	13 (46%)	27 (42%)	26 (53%)	2 (50%)	0.700
Type B Dissection	40 (10%)	6 (8.5%)	9 (5.8%)	24 (16%)	1 (8.3%)	0.030
Acute	26 (65%)	3 (50%)	4 (44%)	18 (75%)	1 (100%)	0.300
Non-Type A non-Type B dissection	44 (11%)	8 (11%)	14 (9.1%)	22 (15%)	0 (0%)	0.300
Acute	28 (64%)	3 (38%)	10 (71%)	15 (68%)	0 (NA%)	0.300
Aortic Aneurysm	120 (31%)	25 (35%)	51 (33%)	40 (27%)	4 (33%)	0.500
Penetrating Aortic Ulcer	27 (7.0%)	2 (2.8%)	10 (6.5%)	13 (8.7%)	2 (17%)	0.200

^1^ Median (Q1, Q3); *n* (%); ^2^ Kruskal–Wallis rank sum test; Fisher’s exact test.

**Table 3:** Surgical details.

Characteristic	BMI					
Number of patients	Overall N = 387 ^1^	≥30 N = 71 ^1^	30 > x ≥ 25 N = 154 ^1^	25 > x ≥ 18.5 N = 150 ^1^	<18.5 N = 12 ^1^	*p*-Value ^2^
Concomitant cardiac or aortic procedure surgery (total)	152 (40%)	34 (48%)	54 (36%)	58 (39%)	6 (55%)	0.200
Valve-carrying conduit	45 (12%)	14 (20%)	14 (9.2%)	14 (9.4%)	3 (27%)	0.034
Valve-sparing aortic root replacement	34 (8.9%)	8 (11%)	10 (6.5%)	15 (10%)	1 (9.1%)	0.500
Aortic valve replacement	58 (15%)	12 (17%)	21 (14%)	23 (15%)	2 (18%)	0.800
Coronary artery bypass grafting	55 (14%)	12 (17%)	22 (14%)	20 (13%)	1 (9.1%)	0.900
Beating-heart procedure	67 (17%)	11 (16%)	26 (17%)	27 (18%)	3 (27%)	0.800
Operation time (min)	381 (327, 451)	400 (339, 479)	371 (324, 434)	379 (326, 435)	363 (335, 384)	0.200
Cardiopulmonary bypass time (min)	211 (178, 252)	226 (177, 275)	212 (182, 247)	206 (175, 251)	208 (159, 232)	0.300
Cross-clamp time (min)	122 (94, 158)	132 (97, 173)	122 (95, 152)	117 (93, 148)	136 (87, 189)	0.300
Selective cerebral perfusion time (min)	100 (70, 128)	102. (83, 129)	106 (69, 139)	96 (63, 115)	103 (76, 113)	0.200
Lowest body temperature during surgery (°C)	24.8 (24.2, 25.4)	24.9 (24.4, 25.6)	24.8 (24.2, 25.4)	24.9 (24, 25.3)	25.1 (24.5, 25.5)	0.500
Packed red blood cells (units)	5 (3, 8)	4 (2, 8.)	5 (3, 8)	6 (4, 9)	7 (4, 9)	0.008
Platelets (units)	3 (2, 4)	3 (2, 4)	3 (2, 4)	3 (2, 4)	2 (2, 4)	0.600
Fresh frozen plasma (units)	7 (4, 10)	7 (4, 10)	7 (4, 11)	6 (4, 10)	6 (5, 16)	0.600
Estimated blood volume in mL	4595.92 (2898.86, 7231.67)	12,001.74 (9827.09, 14,351.40)	5621.93 (4717.27, 6638.81)	2699.12 (2139.74, 3139.19)	3090.56 (2677.27, 3699.27)	<0.001
% of estimated blood volume transfused via PRBC	23.18 (10.46, 43.09)	6.93 (3.09, 11.61)	15.97 (10.78, 27.10)	44.72 (30.79, 72.57)	41.10 (22.45, 54.78)	<0.001
% of estimated blood volume transfused via Platelets	14.19 (7.81, 22.85)	5.37 (4.02, 7.93)	11.46 (8.00, 17.36)	23.16 (16.43, 35.21)	14.56 (12.21, 17.40)	<0.001
% of estimated blood volume transfused via FFP	35.80 (18.55, 56.52)	12.99 (9.57, 23.13)	29.14 (16.72, 43.24)	55.59 (39.80, 86.51)	52.83 (36.41, 71.96)	<0.001
% of estimated blood volume transfused in total	74.14 (39.93, 130.55)	27.85 (19.33, 40.43)	60.38 (39.49, 83.97)	134.45 (96.75, 173.29)	102.49 (86.09, 135.81)	<0.001

^1^ *n* (%); Median (Q1, Q3); ^2^ Fisher’s exact test; Kruskal–Wallis rank sum test.

**Table 4:** Outcome characteristics.

Characteristic	BMI					
Number of patients	Overall N = 387 ^1^	≥30 N = 71 ^1^	30 > x ≥ 25 N = 154 ^1^	25 > x ≥ 18.5 N = 150 ^1^	<18.5 N = 12 ^1^	*p*-Value ^2^
Postoperative stroke	60 (16%)	6 (8.6%)	25 (16%)	29 (20%)	0 (0%)	0.100
Postoperative dialysis	40 (11%)	3 (4.3%)	16 (11%)	21 (14%)	0 (0%)	0.110
Postoperative paraplegia	6 (1.6%)	1 (1.4%)	1 (0.7%)	4 (2.7%)	0 (0%)	0.400
Postoperative tracheotomy	22 (5.8%)	5 (7.1%)	8 (5.3%)	9 (6.1%)	0 (0%)	>0.900
ICU ^3^ stay (days)	6 (3, 10)	6 (3, 10)	5 (3, 9)	6 (4, 12)	9 (4, 13)	0.300
Hospital stay (days)	17 (13, 23)	16 (14, 22)	16 (12, 22)	18 (14, 24)	23 (21, 33)	0.032
In-hospital mortality	54 (14%)	11 (15%)	22 (14%)	19 (13%)	2 (18%)	0.800
New FET thrombus in first follow-up CTA	22 (7.4%)	4 (7.1%)	6 (5.1%)	7 (6.3%)	5 (42%)	0.003
Time to first follow-up CTA (years)	0.25 (0.01, 0.50)	0.37 (0.02, 0.52)	0.27 (0.00, 0.52)	0.21 (0.00, 0.49)	0.10 (0.02, 0.52)	0.600
Follow-up (years)	3.45 (1.47, 5.65)	2.47 (1.60, 4.70)	3.52 (1.44, 5.94)	3.60 (1.44, 5.94)	3.85 (0.79, 5.45)	0.500

^1^ *n* (%); Median (Q1, Q3); ^2^ Fisher’s exact test; Kruskal–Wallis rank sum test; ^3^ Intensive Care Unit.

## Data Availability

The datasets presented in this article are not readily available because of the requirements of our institutional review board. Individual reasonable requests will be evaluated by the corresponding author.

## Abbreviations

The following abbreviations are used in this manuscript:

BMI	Body Mass Index
CTA	Computed Tomography Angiography
FET	Frozen Elephant Trunk
HTAD	Heritable Thoracic Aortic Disease
ICU	Intensive Care Unit
PRBCs	Packed Red Blood Cells
